# THE IMPACT OF A PARO INTERVENTION ON DEPRESSION AND WELL-BEING IN OLDER ADULTS WITH DEPRESSION

**DOI:** 10.1093/geroni/igz038.1236

**Published:** 2019-11-08

**Authors:** Shu-Chuan Chen, Wendy Moyle, Cindy Jones

**Affiliations:** 1 Griffith University, Brisbane, Queensland, Australia; 2 Griffith University, Queensland, Australia; 3 Bond University, Brisbane, Queensland, Australia

## Abstract

Aim: This study aimed to explore the effect of a social robot Paro intervention on depression and well-being in older adults with depression living in long-term care facilities in Taiwan. Methods: This study was adopted a single group and quasi-experimental with repeated measures design. Each participant participated in two stages: observation and Paro intervention stages. Stage 1 was an 8-week observation stage in long-term care facilities where the purpose was to observe the normal mood, behaviour and activities of older adults with depression. In stage 2, each participant was given a Paro by the researcher to keep for 24 hours for 7 days in for 8 weeks. Outcome measurements were obtained 4 times: a week before the intervention (T1), immediately the end of 8-week observation (T2), mid-point of Paro intervention (T3), and immediately the end of 8-week Paro intervention (T4). Instruments included the Geriatric Depression Scale, the UCLA Loneliness Scale version 3, and the World Health Organization Quality of Life Questionnaire-OLD. Results: There were 20 participants completed the study. The mean age of participants was 81.1years (SD = 8.2). After 8-week Paro intervention, statistically significant differences in changes were found on depression, loneliness, and quality of life from pre-intervention to post-intervention. Conclusion: This study was found that Paro intervention has beneficial effects on depression and mental well-being for older people with depression in long-term care facilities. Paro Intervention might be a suitable psychosocial intervention for older people with depression and should be considered as a useful tool in clinical practice.

